# Draft Genome Sequences of Two Novel *Pseudomonas* Strains Exhibiting Differential Hypersensitivity Reactions on Tobacco and Corn Seedlings

**DOI:** 10.1128/genomeA.01057-16

**Published:** 2016-10-06

**Authors:** Caetanie Fometeu Tchagang, Renlin Xu, Shima Mehrtash, Shabnam Rahimi, Aïssata Sidibé, Xiang Li, Eden S. P. Bromfield, James Tabi Tambong

**Affiliations:** a Biodiversity, Environmental Health Program, Ottawa Research and Development Centre (ORDC), Agriculture and Agri-Food Canada, Ottawa, Ontario, Canada; b La Cité Collégiale, Ottawa, Ontario, Canada; c Canadian Food Inspection Agency, Charlottetown, Prince Edward Island, Canada

## Abstract

Two novel *Pseudomonas* strains (S1E40 and S3E12) isolated from corn roots are antagonistic to *Rhizoctonia solani* and exhibit differential hypersensitivity reactions on tobacco and corn seedlings. We report here the draft genome sequences of strains S1E40 and S3E12, consisting of 6.98 and 7.06 Mb with 6,150 and 6,129 predicted protein-coding sequences, respectively.

## GENOME ANNOUNCEMENT

*Pseudomonas* strains isolated from corn roots previously drenched with a 10% soil suspension were characterized by sequencing the 16S rRNA and three housekeeping genes (*gyrB*, *rpoB*, and *rpoD*), Biolog carbon utilization, and hypersensitivity reactions on tobacco and corn seedlings. Strains S1E40 and S3E12 are identified as potential novel species within the genus *Pseudomonas*. Both strains are antagonistic (*in vitro*) to a fungal plant pathogen, *Rhizoctonia solani*. However, these novel strains exhibit differential hypersensitivity reactions (HRs) on tobacco and corn seedlings. Strain S3E12 induces HRs on tobacco and corn seedlings, but strain S1E40 does not. We report here the draft genome sequences of *Pseudomonas* strains S1E40 and S3E12. The draft genome sequences were determined by paired-end sequencing using Illumina MiSeq technology (Génome Québec, Montreal, Canada). A total of 4,290,298 and 4,472,574 paired-end reads, each 250 bp in length, for strain S1E40 and S3E12, respectively, were generated. The quality of the reads was checked using FastQC version 0.11.3 (1). *De novo* assemblies were performed using ABySS version 1.5.2 (2) at k-mer values of 75 to 113. k-mer values of 85 and 95 gave the best assembly, producing 49 and 38 scaffolds after the discard of scaffolds with length <300 bp for strains S1E40 and S3E12, respectively. The total size of the draft genome is 6,984,066 bp (minimum, 402 bp; maximum, 834,588 bp; *N*_50_, 206,686 bp) and 7,062,659 bp (minimum, 303 bp; maximum, 935,416 bp; *N*_50_, 420,043 bp) for S1E40 and S3E12, respectively. The G+C content of the draft genomes is 61.6% and 60.9%, with overall estimated coverage of 153× and 158× for strains S1E40 and S3E12, respectively.

The NCBI Prokaryotic Genome Automatic Annotation Pipeline (PGAAP) revealed that the draft genome of strain S1E40 contains 6,150 predicted protein-coding sequences, 61 tRNAs, four noncoding RNAs (ncRNAs), 63 pseudogenes, and six, three, and three (5S, 16S, and 23S, respectively) complete rRNAs. Strain S3E12 has 6,129 predicted protein-coding sequences, 79 tRNAs, four ncRNAs, 93 pseudogenes, and seven, 11, and 11 (5S, 16S, and 23S, respectively) complete rRNAs. Compared to PGAAP, RAST (3) predicted 6,218 and 6,191 coding sequences for strains S1E40 and S3E12, with 73 and 113 RNAs, respectively. The number of predicted rRNA genes was confirmed by RNAmmer version 1.2 (4), as implemented in CMG-BioTools (5). Strains S1E40 and S3E12 share 4,484 protein families, with 1,260 and 1,342 unique families, respectively, and each possesses 10 antibiotic resistance genes based on the PATRIC analysis tool (6). A more detailed analysis of these draft genomes will provide insight into why strain S3E12, but not strain S1E40, induces hypersensitivity on tobacco and corn seedlings.

### Accession number(s).

This whole-genome shotgun project has been deposited at DDBJ/EMBL/GenBank under the accession numbers MAUE00000000 (strain S1E40) and MBDT00000000 (strain S3E12). The versions described in this paper are the first versions, MAUE01000000 and MBDT01000000, respectively.
